# Beyond *Q*: The Importance of the Resonance Amplitude for Photonic Sensors

**DOI:** 10.1021/acsphotonics.2c00188

**Published:** 2022-04-15

**Authors:** Donato Conteduca, Guilherme S. Arruda, Isabel Barth, Yue Wang, Thomas F. Krauss, Emiliano R. Martins

**Affiliations:** † Photonics Group, School of Physics, Engineering and Technology, University of York, Heslington, York YO10 5DD, U.K.; ‡ São Carlos School of Engineering, Department of Electrical and Computer Engineering, University of São Paulo, São Carlos-SP 13566-590, Brazil

**Keywords:** resonance amplitude, photonic sensors, limit of detection, metasurface, dielectric resonator, figure of merit

## Abstract

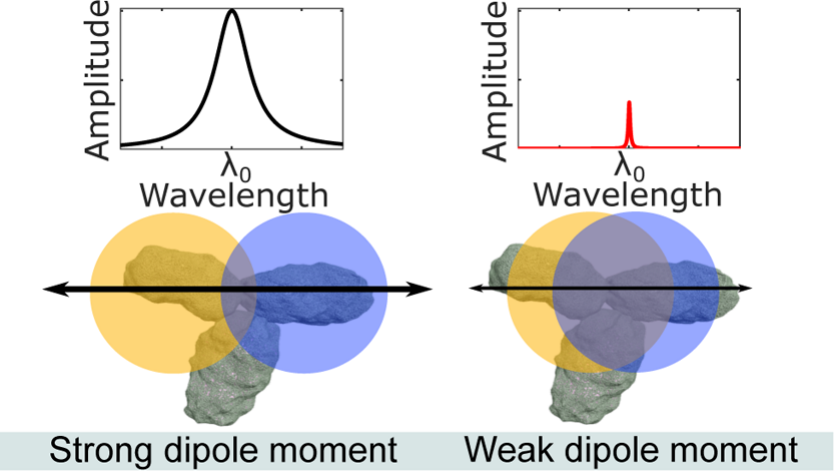

Resonant photonic sensors are enjoying much attention based on the worldwide drive toward personalized healthcare diagnostics and the need to better monitor the environment. Recent developments exploiting novel concepts such as metasurfaces, bound states in the continuum, and topological sensing have added to the interest in this topic. The drive toward increasingly higher quality (*Q*)-factors, combined with the requirement for low costs, makes it critical to understand the impact of realistic limitations such as losses on photonic sensors. Traditionally, it is assumed that the reduction in the *Q*-factor sufficiently accounts for the presence of loss. Here, we highlight that this assumption is overly simplistic, and we show that losses have a stronger impact on the resonance amplitude than on the *Q*-factor. We note that the effect of the resonance amplitude has been largely ignored in the literature, and there is no physical model clearly describing the relationship between the limit of detection (LOD), *Q*-factor, and resonance amplitude. We have, therefore, developed a novel, ab initio analytical model, where we derive the complete figure of merit for resonant photonic sensors and determine their LOD. In addition to highlighting the importance of the optical losses and the resonance amplitude, we show that, counter-intuitively, optimization of the LOD is not achieved by maximization of the *Q*-factor but by counterbalancing the *Q*-factor and amplitude. We validate the model experimentally, put it into context, and show that it is essential for applying novel sensing concepts in realistic scenarios.

## 1 Introduction

Photonic sensors are an important class of sensing devices that use light to detect modifications in an environment.^1^ Exploiting photonic resonances allows such sensors to operate as a label-free modality, which is particularly beneficial for low-cost realizations as required for near-patient testing and environmental monitoring.^2,3^ A key property of such sensors is the limit of detection (LOD), which is the minimum change in the measurand that can be detected by the sensor.^4^ There has been a recent revival in novel photonic structures for sensing based on exciting concepts such as metasurfaces,^5,6^ bound states in the continuum (BIC),^7,8^ and topological sensing.^9,10^ Unlike conventional sensing modalities that were predominantly based on guided-wave optics such as microring resonators,^11^ these new concepts exploit leaky modes; consequently, their ability to achieve high quality (*Q*) factors is more susceptible to scattering and absorption losses. Hence, a model that takes losses into account is required, especially for describing the impact of losses on the resonance amplitude and for exploiting these novel sensing concepts to their maximum potential.

In a milestone paper, White and Fan previously introduced some of the key parameters, such as the *Q*-factor of the resonance (*Q*_R_), its sensitivity to refractive index changes, and the signal-to-noise ratio (SNR) of the system.^12^ Their model, however, did not explicitly consider the losses of the photonic structure and the amplitude of the photonic resonance, which are essential for describing the novel structures referred to above.

Other works have already considered the effect of optical losses on the sensing performance^13^ or highlighted the role of the SNR, confirming that the best sensing performance is not always obtained with the highest possible *Q*-factor.^14−16^ However, a rigorous analytical approach that includes the parameters affecting the LOD of a resonant biosensor and describes their relationship in a closed form is still missing.

**Figure 1:**
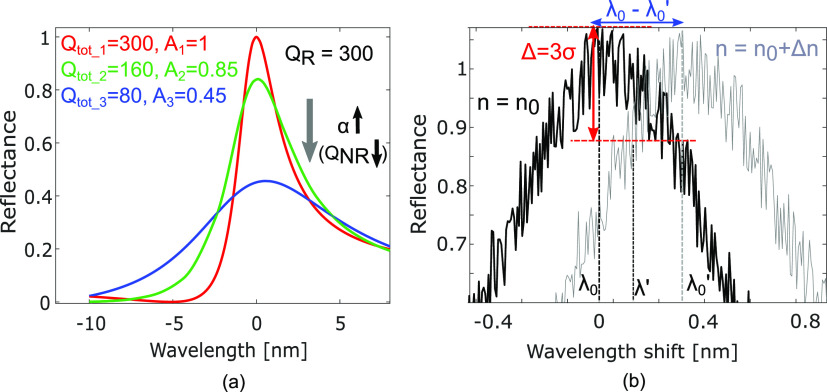
(a) Example of reflectance spectra with same *Q*_R_, but different *Q*_NR_ and resonance amplitude *A*(λ_0_) due to a corresponding increase of the optical losses. (b) Typical experimental reflectance spectrum with noise with *n* = *n*_0_ (black curve) and *n* = *n*_0_ + Δ*n* (gray curve) with *A*(λ_0_) – *A*(λ′) = 3σ.

Here, we investigate in detail the role of the resonance amplitude in the LOD and key design strategies to be followed for optimizing the LOD in lossy systems. We take an a priori approach and use temporal coupled mode theory (TCMT)^17^ to derive the LOD from first principles. We reach a closed form expression that is very instructive for understanding the operation and limitation of resonant photonic sensors. The model is convenient in that it only requires information that is readily accessible to an experimentalist. Taking the resonance amplitude into account, the model highlights the trade-off between the *Q*-factor and losses, losses being highly relevant as they are almost unavoidable in real-world experimental systems, such as BIC resonances that are currently being considered by many research groups worldwide.^18−20^ It is evident from Figure 1a, as an example, how an ideal loss-less resonator presents a *Q*_R_ and resonance amplitude *A* = 1, while for real conditions, the losses, described by the equivalent *Q*-factor, *Q*_NR_ (non-resonant *Q*-factor, higher losses correspond to lower *Q*_NR_), strongly affect both the total *Q*-factor (*Q*_tot_ < *Q*_R_) and the resonance amplitude (*A* < 1).

We show that, in the presence of losses, the LOD is inversely proportional to the product of the resonance peak amplitude and the *Q*_R_. The model also shows that the optimum LOD is reached when *Q*_R_ = *Q*_NR_, which is a manifestation of the well-known critical coupling condition.^7^ Surprisingly, we find that it is more important to improve the amplitude than to reduce the noise of the system, so that the SNR is no longer a key parameter describing the system’s performance. To test the validity of our model, we fabricate a photonic sensor based on guided mode resonances (GMR) and find an excellent agreement between our model and the experimental LOD. Furthermore, we also demonstrate a good match with the model for a microring resonator, which confirms the versatility of the model for optical sensors irrespective of the *Q*-factor.

## 2 Results

### 2.1 Model for LOD

We begin by considering Lorentzian resonances and later extend the discussion to include Fano resonances. A typical photonic resonance is described by TCMT,^18^ assuming that the resonance is coupled to two channels: light couples into the resonance through channel 1 and leaks out through channel 1 (reflection) and channel 2 (transmission). In the case of a Lorentzian resonance, the amplitude can be expressed as follows^7,21^
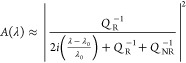
1where *A*(λ) is the signal amplitude as a function of wavelength λ, with λ_0_ being the resonance wavelength; *Q*_R_ is the resonant *Q*-factor, which is the *Q* without losses; and *Q*_NR_ is the non-resonant *Q*-factor that describes both scattering and/or absorption losses. In the absence of losses, *Q*_NR_ = ∞.

The presence of losses is usually associated with a broadening of the resonance, described by the well-known relation *Q*_tot_^–1^ = *Q*_R_^–1^ + *Q*_NR_^–1^. The impact of losses on the amplitude, however, is even more severe than on *Q*. Indeed, eq 1 describes the amplitude of the resonance peak as follows
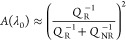
2which shows that the amplitude scales as the square of the *Q*-factor ratio and not linearly. Thus, losses have a stronger impact on the amplitude than on the *Q*-factor, as illustrated in Figure 1a (see also Supporting Information 1).

For every sensor in the real world, the pure Lorentzian of eq 1 is perturbed by noise, as illustrated in Figure 1b. It is the presence of noise that imposes a limit on the minimum change in the measurand that can be detected. It is widely accepted that for a signal to be detectable, it must be at least three times larger than the standard deviation of the noise σ.^12^

As shown in Figure 1b, for a Lorentzian resonance, we define the “signal” as the variation of the resonance amplitude around the reference resonance wavelength λ_0_ caused by a perturbation in the external environment. Notice that the perturbation causes a shift of the resonance wavelength from its reference value λ_0_ to a new value λ_0_^′^, thus changing the amplitude at λ_0_.

We then define the minimum detectable amplitude variation as 3σ, where σ is the standard deviation of the amplitude. Thus, the wavelength λ′ for which a shift of 3σ is obtained satisfies
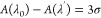
3where *A*(λ_0_) is the resonance amplitude. As illustrated in Figure 1, the wavelength deviation is thus Δλ = λ′ – λ_0_. From eq 1 and 3, the minimum detectable wavelength deviation is (see Supporting Information 2 for the full derivation)
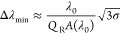
4

The minimum wavelength variation can now be easily correlated with the minimum detectable change in the measurand through the sensitivity *S*, which, by definition, is the ratio of the wavelength shift to the variation of the quantity of interest. Thus, the LOD is given by
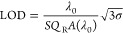
5

In a refractive index sensor, the sensitivity is defined as wavelength shift versus refractive index change *S* = Δλ/Δ*n*, in units of nm/RIU; consequently, the LOD is typically expressed as the minimum detectable refractive index change Δ*n*.

As expected, the LOD depends inversely on *Q*_R_. This dependence is well understood and has driven sensor research toward resonances with increasingly large *Q*-factor.^22,23^ However, our model highlights that this effort is only justified in ideal scenarios, without losses, because, in the presence of losses, the resonance peak *A*(λ_0_) also depends on *Q*_NR_ (eq 2).

It is then instructive to compare the model obtained in eq 5 with the widely used figure of merit *SQ*_tot_, where *Q*_tot_ is the total measured *Q*. Substituting eq 2 into 5, and recalling that *Q*_tot_^–1^ = *Q*_R_^–1^ + *Q*_NR_^–1^, we find
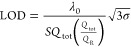
6

Thus, according to eq 6, the impact of losses on the amplitude introduce a correction factor of *Q*_tot_/*Q*_R_ on the traditional figure of merit *SQ*_tot_. The significance of the correction factor *Q*_tot_/*Q*_R_ is expected to increase as *Q*-factors are enhanced, especially in low-cost resonators, which tend to exhibit higher losses.

From eq 5, the minimum LOD is reached when the product *Q*_R_·*A*(λ_0_) is maximized. We emphasize that, depending on the relationship between *Q*_R_ and *Q*_NR_, a larger *Q*_R_ may result in a worse LOD. Indeed, it is a straightforward matter to prove that, for a given *Q*_NR_, the product *Q*_R_·*A*(λ_0_) is maximized when *Q*_R_ = *Q*_NR_, which is the critical coupling condition^24^ (see Supporting Information 3). Therefore, our model shows that losses set a limit on the optimum *Q*-factor for sensing, a condition that is largely ignored in the literature.

We also note that losses impact more the resonance amplitude than the *Q*-factor, which is apparent from the fact that the term *Q*_R_, *Q*_NR_ in eq 2 is squared, whereas the corresponding expression for the total *Q*-factor is not (for more details, see (Supporting Information 1 and 3). This insight also supports our strategy of optimizing the product *Q*_R_·*A*(λ_0_) in the presence of loss, instead of maximizing any one parameter individually, or not taking losses into account.

In addition, our model highlights a counter-intuitive relationship between the signal and noise. It is a common perception that a system’s performance is dependent only on the SNR, so that increasing the signal and noise proportionally does not affect the system performance. Such a perception is also captured by White and Fan,^12^ whose fitted equation for the LOD depends solely on the SNR. This is not true for resonant sensors suffering from losses, however. Instead, the signal and noise weigh differently in the equation for the LOD (eq 5), so that it is more important to improve the signal (the resonance amplitude) than to reduce the noise. For example, according to eq 5, if the resonance amplitude *A*(λ_0_) and the noise σ are both doubled, the LOD will be reduced by a factor of . This surprising feature is a consequence of resonance reshaping: reducing the losses not only increases the amplitude but also reshapes the resonance. For a more detailed explanation, see Supporting Information 4.

### 2.2 Validation of the Model

**Figure 2:**
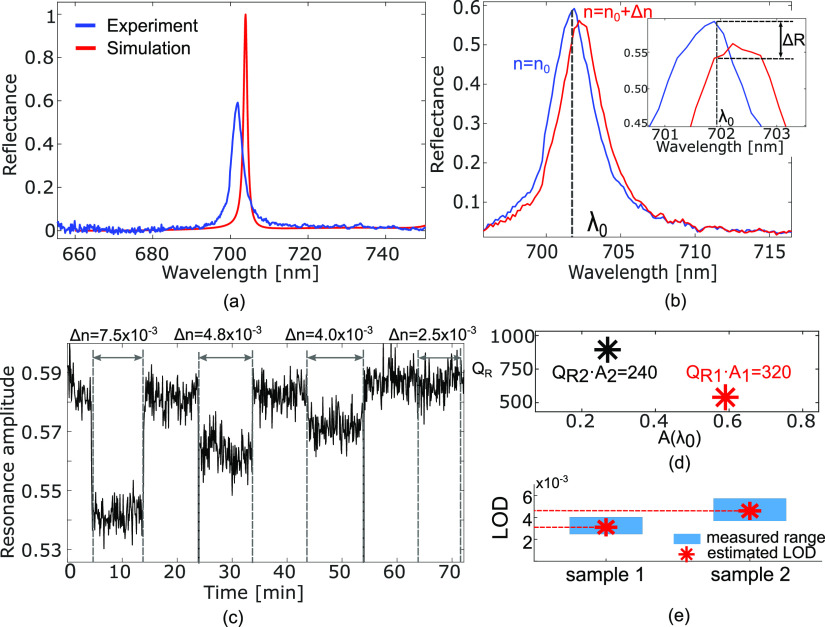
(a) Experimental (blue curve) and simulated (red curve) spectra of the GMR structure. (b) Reflectance spectrum with *n* = *n*_0_ (blue curve) and *n* = *n*_0_ + Δ*n* (red curve), assuming *n*_0_ as the water refractive index (= 1.3329) and Δ*n* = 7.5 × 10^–3^. (c) Resonance amplitude change over time with different values of refractive index of the solution with the sensor in (a). (d) *Q*_R_ vs *A*(λ_0_) for the GMR structure in (a) (sample 1) compared to another GMR sensors with a different value of *Q*_R_·*A* (sample 2) and (e) corresponding expected LOD and measured range of refractive index change Δ*n*.

**Figure 3:**
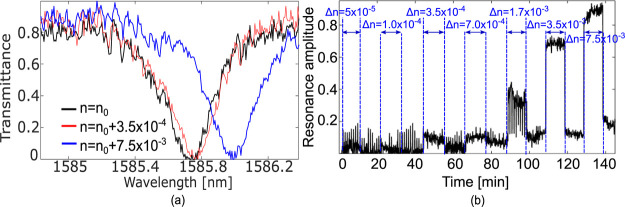
(a) Transmission spectra and (b) resonance amplitude change of a microring resonator in the Silicon on Insulator (SOI) technology^27^ for different refractive index values of the surrounding medium with *n*_0_ = 1.31. Please note that the refractive index values are not equally spaced, hence the curve appears non-linear.

To validate the model and to exemplify its use, we fabricated a photonic sensor and determined the LOD experimentally. In our example, the sensor is based on a GMR,^25^ but we emphasize that the model is general and can be applied to any resonant sensor [note that we also exemplify the model for microring resonators (see Figure 3)]. Figure 2a shows two Lorentzian resonances: the blue curve describes the experimentally measured resonance, while the red curve represents the simulated one. It is obvious that the experimental resonance is subject to the scattering (and possibly also absorption) losses that are present in any real system. Accordingly, the peak value is not unity, and the resonance linewidth is broader than the linewidth of the simulated curve. In our model, the peak value corresponds to the *A*(λ_0_) parameter and is 0.59 for this particular example. The simulated resonance, on the other hand, does not include any losses and it is not affected by limitations in the optical setup, for example, the spectral resolution of the spectrometer, which instead impacts the resonance amplitude. Therefore, the *Q*-factor of the simulated resonance is *Q*_R_ of the model, in our example *Q*_R_ = 540.

The next parameter we consider is the sensitivity *S*. We experimentally determine a bulk sensitivity for our sensor of *S* = 84 nm/RIU (see Supporting Information 5). We also measured the standard deviation of the amplitude noise and found it to be 3σ = 1.42 × 10^–2^. The 3σ value has been determined by evaluating the deviation of the signal amplitude monitored at a fixed wavelength (λ_0_) over 15 min, while keeping the refractive index of the solution constant. By using these values in our model (eq 5), we find that our model predicts an LOD of 3.1 × 10^–3^ RIU.

This calculation determines the LOD from the model, using parameters that are easily accessed by the experimentalists. To verify the model experimentally, we measured the change in resonance amplitude for different refractive indices, as shown in Figure 2b. As it is clear from Figure 2c, we can clearly discriminate the step change in the signal for Δ*n* = 4 × 10^–3^ RIU, but we cannot do so for Δ*n* = 2.5 × 10^–3^ RIU. By interpolating the experimental shift as a function of the refractive index change, the expected LOD can be extrapolated by the intersection of the curve with the 3σ value, which corresponds to an LOD of 3.0 × 10^–3^ RIU, with a mismatch between the model result and the experiments of only 3.2%, confirming the accuracy of the model for the LOD prediction (see Table 1). For comparison, we then used the same experimental parameters with the model determined empirically in ref (12) and found that their model predicts an LOD of 2.1 × 10^–3^ RIU, underestimating the value measured experimentally. One may argue that this difference in LOD is not very large and therefore not significant in the context of the experimental uncertainty, but as we show below, our model is also better at describing important trends in sensor design.

**Table 1:** Comparison between the Experimental LOD and the Expected Values Obtained from the Model for Resonant Structures with Different Values of *Q*_R_·*A*(λ_0_)

	*Q*_R_·*A*(λ_0_)	estimated experimental LOD [RIU]	expected LOD from the model [RIU]	model uncertainty (%)
GMR sensor 1 (low-Q)	3.2 × 10^2^	3.0 × 10^–3^	3.1 × 10^–3^	3.2
GMR sensor 2 (moderate-Q)	2.4 × 10^2^	4.9 × 10^–3^	4.6 × 10^–3^	6.5
microring (high-Q)	2.1 × 10^4^	2.62 × 10^–4^	2.9 × 10^–4^	9.6

Accordingly, to further validate our model and to highlight the important dependence of the LOD on the product *Q*_R_·*A*(λ_0_), we consider a similar GMR structure to that described in Figure 2a (See Supporting Information 6), but now with a higher *Q*-factor, yet with a lower product *Q*_R_·*A*(λ_0_). Specifically, we now have *Q*_R_ = 890 and *A*(λ_0_) = 0.27, thus resulting in *Q*_R_·*A*(λ_0_) = 240.3. This product is lower than that in the first experiment (earlier, we had in *Q*_R_·*A*(λ_0_) = 318.6). According to our model, the lower product results in a worse LOD than before. Indeed, using these numbers in eq 5 (the resonance wavelength is now λ_0_ = 743 nm), one finds LOD = 4.6 × 10^–3^ RIU (Figure 2e). By repeating the experiment with this new resonance, we find that the experimental LOD now lies between 3.7 × 10^–3^ RIU and 5.6 × 10^–3^ RIU, with a predicted value of 4.9 × 10^–3^ RIU, with an inaccuracy of 6.5% between the model prediction and the experiments (See Supporting Information 6, Table 1). Thus, our experiment confirms that due to the inherent losses of the system, a higher *Q*-factor results in a worse LOD, when the product *Q*_R_·*A*(λ_0_) is lower (Figure 2d).

In order to further demonstrate the versatility and generality of our model, we also consider a microring resonator with a relatively high *Q*-factor, that is, more than 1 order of magnitude higher than the previous sensors based on GMR. More details about the design and fabrication of the microring resonator are reported in refs (26) and (27). The sensor exhibits *A*(λ_0_) ∼ 0.8 with λ_0_ = 1585.8 nm (Figure 3a), while the simulated *Q*-factor is *Q*_R_ ∼ 2.6 × 10^4^. We have measured a noise level of 3σ = 0.06 and a sensitivity of 65 nm/RIU. By using these values in our model, we predict an LOD = 2.9 × 10^–4^ RIU (Table 1). The experiments verify an LOD that lies between 1 × 10^–4^ RIU and 3.5 × 10^–4^ RIU (Figure 3b), with an expected LOD value of 2.62 × 10^–4^ RIU, validating the predicted value from the model with an uncertainty less than 10%. The fact that the model prediction is also correct for high *Q*-factor cavities confirms its generality.

A widespread strategy to improving the LOD is to apply fitting methods to track the resonance shift.^8,27−30^ Beyond clarifying the dependence of the LOD on the various parameters of the resonance, our model can also be used to quantify the effect of such fitting methods. As an example, we use the method to compare the LOD obtained with and without fitting.

Using this method, we can now infer the equivalent value of 3σ from the model that would be required to reach such a low LOD without fitting. Using eq 4, we find that the equivalent noise is up to 3 orders of magnitude lower than the raw noise, confirming that a simple fitting procedure can provide a comparable LOD that would be obtained in a system with a standard deviation of the noise that is a thousand times lower (See Supporting Information 7).

### 2.3 Fano Resonances

**Figure 4:**
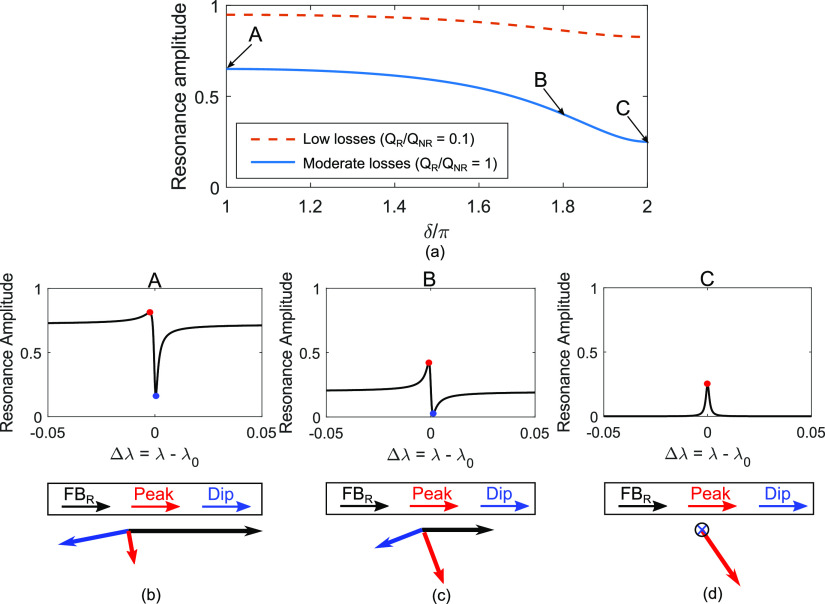
Resonance amplitude as a function of the phase of the FP background δ for the case of moderate losses with *Q*_R_/*Q*_NR_ = 1 (blue curve) and low losses with *Q*_R_/*Q*_NR_ = 0.1 (red-dotted curve). Reflectance spectra with (b) δ/π = 1 and (c) δ/π = 1.8 and related phase relationships between the FP background (black arrow) and the cavity resonance peak (red arrow) and dip (blue arrow), and (d) with δ/π = 2.

So far, we have used only Lorentzian resonances. To emphasize the generality of our model, we now show that it can be extended to describe Fano resonances as well. The main difference is that, for Fano resonances, the dynamic range (DR), defined as the difference between the peak and dip,^31^ plays the role of the resonance amplitude *A*(λ_0_). One interesting feature of Fano resonances is that, in the presence of losses, the DR depends on the phase of the Fabry–Perot (FP) background resonance. This feature is shown in Figure 4a for two different regimes: a low loss regime (*Q*_R_ = 10^3^ and *Q*_NR_ = 10^4^ with *Q*_R_/*Q*_NR_ = 0.1) and a moderate loss regime with *Q*_R_ = *Q*_NR_ = 10^3^ (*Q*_R_/*Q*_NR_ = 1). The horizontal axis shows the phase of the FP background resonance, for which δ = 2π is the condition for a Lorentzian resonance (see Supporting Information 8 for more details).^32^ As the phase is decreased from the Lorentzian value and into the Fano region, the DR increases, and it can even double in the high loss regime. To illustrate the origin of this behavior, resonances for three different phases δ are shown in Figure 4. The phase relationships between the FP background and the cavity resonance are illustrated by the arrows in the insets of Figure 4b,c,d. Notice in the insets in Figure 4b,c that the blue arrows, which represent the phase and amplitude at the dip, are nearly antiparallel to the black arrows, which represent the FP background. On the other hand, the red arrows, representing the peak, are roughly orthogonal to the FP background (black arrows). Therefore, the phase relationships are more favorable to destructive interference, which leads to the formation of the dips, rather than to constructive interference, which instead leads to the formation of the peaks (Supporting Information 8). Combined with our model, these results indicate that it is possible to improve the LOD of a sensor by a factor of 2, for a fixed *Q*_NR_, by properly adjusting the phase of the background resonance. In practice, the adjustment of the background phase depends on the type of resonance; for GMR, the FP background depends on the thickness of the waveguide film and on its refractive index.^33^

## 3 Conclusions

We have developed a simple and intuitive model to describe the LOD of resonant photonic sensors in terms of resonance parameters that are readily accessible for the experimentalists and that include losses and their impact on the resonance amplitude. The model is extremely timely as many novel sensing concepts such as metasurfaces, BICs, and topological sensing rely on leaky modes that are more susceptible to losses than previous concepts based on guided modes. Our model is derived from first principles and is based on the temporal coupled mode theory, with no assumptions other than that the system can be described by the general condition of a single resonance coupled to two channels. Our key finding is that the widely used figure of merit, which multiplies sensitivity and the *Q*-factor of the resonator, is overly simplistic because it considers neither the losses nor the amplitude of the resonance. Instead, our model brings out the requirement that the LOD is optimized when the product *Q*_R_·*A*(λ_0_) is maximized. This requirement shows that an exceedingly large *Q*-factor may lead to a worsening of the LOD, contrary to a widely held belief in the community. Indeed, for a given loss, the LOD is optimized by the critical coupling condition between the intrinsic resonant quality-factor *Q*_R_ and the loss quality factor *Q*_NR_. Thus, our model allows the prediction of the LOD that can be obtained in a realistic system, where all the required information can be gathered by straightforward inspection of the simulation and the experimental resonances. We have validated our model experimentally and have used it to show the benefit of simple data processing strategies such as fitting procedures. Finally, we have shown that it is possible to improve the LOD up to a factor of 2 by entering the Fano regime and judiciously adjusting the background resonance phase. Our model rigorously clarifies the effect of losses on resonators typically used for photonic sensing and it clarifies which parameters are most relevant for the further improvement of resonant photonic sensors, in particular the surprising insight that resonance amplitude is more important than SNR.

## Supporting Information Available

A detailed derivation of the analytical model for the LOD, together with additional experimental results on the sensitivity of the guided mode resonance sensors, effects of the fitting methods on the LOD, and the impact of Fano-shaped resonances (PDF)

## Notes

Data availability: Data underlying the results presented in this paper are not publicly available at this time, but may be obtained from the authors upon reasonable request.
