# Supplementary material for: Beyond Q: The Importance of the Resonance Amplitude for Photonic Sensors
Source: ACS Photonics. 2022 Apr 15;9(5):1757–63. doi: 10.1021/acsphotonics.2c00188 (PMC9121374; doi:10.1021/acsphotonics.2c00188)
Supplement: Supplementary file 1 — ph2c00188_si_001.pdf [file ph2c00188_si_001.pdf]

Supplementary Information

Beyond Q: The importance of the resonance amplitude for photonic sensors

DONATO CONTEDUCA[1*], GUILHERME S. ARRUDA[2], ISABEL BARTH[1], YUE WANG[1], THOMAS F. KRAUSS[1] AND EMILIANO R MARTINS[2]

[1] *Photonics Group, School of Physics, Engineering and Technology, University of York, Heslington, York YO10 5DD, UK*
[2] *São Carlos School of Engineering, Department of Electrical and Computer Engineering, University of São Paulo, São Carlos-SP, 13566-590, Brazil*

**e-mail: donato.conteduca@york.ac.uk*

Supplementary Information 1

Dependence of the Q-factor and resonance amplitude on the optical losses (Q_R/Q_{NR})

In this section, we show that impact of losses is more severe on the resonance amplitude than on the Q factor.

The losses can be conveniently expressed in terms of the ratio Q_R/Q_{NR}. According to eq. (5) of the main manuscript, the resonance amplitude depends on this ratio as:

$$A(\lambda_0) = \left(\frac{1}{1+\frac{Q_R}{Q_{NR}}} \right)^2 \quad \text{(S18)}$$

The total quality factor, on the other hand, is given by:

$$Q_{tot} = \frac{Q_R Q_{NR}}{Q_R + Q_{NR}} = Q_R \frac{1}{1+\frac{Q_R}{Q_{NR}}} \quad \text{(S19)}$$

Thus:

$$\frac{Q_{tot}}{Q_R} = \frac{1}{1+\frac{Q_R}{Q_{NR}}} \quad \text{(S20)}$$

Comparing eq. (S18) with eq. (S20), it is apparent that the resonance amplitude decreases as the square of the ratio between Q_{tot} and Q_R. Thus, for example, if the losses reduce the Q by a factor of 2, then the amplitude will be reduced by a factor of 4. This square dependence explains why the impact of losses is more severe on the resonance amplitude than on the Q-factor. This effect is illustrated in figure S1.

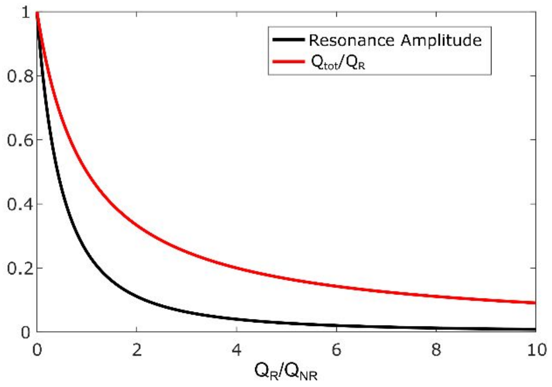

Figure S1. Dependence of the Q-factor (red curve) and resonance amplitude (black curve) on the optical losses, expressed as the ratio Q_R/Q_{NR}.

Supplementary Information 2

Derivation of the model

For the model derivation, we define a relationship for the limit of detection that takes into account the resonance amplitude. We start by defining the minimum detectable amplitude variation as:

$$A(\lambda_0) - A(\lambda') = 3\sigma \qquad \text{(S1)}$$

From temporal coupled mode theory, assuming a symmetric system, one has [18]

$$A = \left| r - f(\lambda) \right|^2 \qquad \text{(S2)}$$

Where r is the reflection coefficient of the Fabry-Perot background and $f(\lambda)$ is defined as follows:

$$f(\lambda) \approx \left| \frac{Q_R^{-1}}{2i\left(\frac{\lambda - \lambda_0}{\lambda_0}\right) + Q_R^{-1} + Q_{NR}^{-1}} \right|^2 \qquad \text{(S3)}$$

At this point, for simplicity, we assume that the resonance is Lorentzian in nature (the case of a Fano resonance is discussed in Supplementary Information 6). The Lorentzian condition is obtained when the Fabry-Perot background is at a minimum, which corresponds to $r = 0$. In this case, the signal amplitude from eq. (S2) simplifies as follows,

$$A(\lambda) = \left| f(\lambda) \right|^2 \qquad \text{(S4)}$$

And on resonance:

$$A(\lambda_0) = \left| f(\lambda_0) \right|^2 = \left| \frac{Q_R^{-1}}{Q_R^{-1} + Q_{NR}^{-1}} \right|^2 = \frac{1}{\left(1 + \frac{Q_R}{Q_{NR}}\right)^2} \qquad \text{(S5)}$$

Now, we define *x* as:

$$x = (\frac{\lambda' - \lambda_0}{\lambda_0}) \tag{S6}$$

So we obtain:

$$A(\lambda_0) - |f(x)|^2 = 3\sigma \tag{S7}$$

Then eq. (S1) becomes:

$$\left| \frac{1}{2ixQ_R + 1 + \frac{Q_R}{Q_{NR}}} \right|^2 = A(\lambda_0) - 3\sigma \tag{S8}$$

Which corresponds to:

$$\frac{1}{4x^2 Q_R^2 + \left(1 + \frac{Q_R}{Q_{NR}}\right)^2} = A(\lambda_0) - 3\sigma \tag{S9}$$

and from the definition of $A(\lambda_0)$ in eq. (S5), eq. (S9) can be expressed as:

$$\frac{1}{4x^2 Q_R^2 + A(\lambda_0)^{-1}} = A(\lambda_0) - 3\sigma \tag{S10}$$

Rearranging the terms,

$$4x^2 Q_R^2 (A(\lambda_0) - 3\sigma) + 1 - \frac{3\sigma}{A(\lambda_0)} = 1 \tag{S11}$$

And therefore:

$$x^2 = \frac{3\sigma}{4A(\lambda_0)(A(\lambda_0) - 3\sigma)Q_R^2} \tag{S12}$$

From which we find:

$$x = \frac{1}{2Q_R}\sqrt{\frac{3\sigma}{A(\lambda_0)(A(\lambda_0) - 3\sigma)}} \tag{S13}$$

The minimum wavelength shift that is detectable is $\Delta\lambda_{min} = 2(\lambda' - \lambda_0)$. Thus, from eq. (S6), we have:

$$x = \frac{\Delta\lambda_{\min}}{2\lambda_0} \tag{S14}$$

Equation (S13) can then be expressed as:

$$\Delta\lambda_{\min} = \frac{\lambda_0}{Q_R}\sqrt{\frac{3\sigma}{A(\lambda_0)(A(\lambda_0) - 3\sigma)}} \tag{S15}$$

Assuming a resonance amplitude that is much larger than the system noise ($A(\lambda_0) >> 3\sigma$), the previous equation becomes:

$$\Delta\lambda \approx \frac{\lambda_0}{A(\lambda_0)Q_R}\sqrt{3\sigma} \qquad \text{(S16)}$$

Which is eq. (4) of the main manuscript.

Supplementary Information 3

Derivation of the optimum condition for the minimum LOD

According to eq (4) of the main manuscript, the LOD is optimized when the product $Q_R A(\lambda_0)$ is maximized. For a given system, with the loss fixed (fixed Q_{NR}), this product is a function of Q_R:

$$f(Q_R) = Q_R A(\lambda_0) = Q_R\left(\frac{Q_R^{-1}}{Q_R^{-1}+Q_{NR}^{-1}}\right)^2 \qquad \text{(S17)}$$

where eq. (1) was used to evaluate $A(\lambda_0)$.

The optimum LOD is found at the point of maximum of $f(Q_R)$. By setting $df(Q_R)/dQ_R = 0$, one readily finds that the maximum of $f(Q_R)$ is obtained when $Q_R = Q_{NR}$, which is the critical coupling condition.

Supplementary Information 4

The effect of the resonance amplitude for the LOD

The role of resonance reshaping can be conveniently described by inspection of Equation 2: naively, one may argue that increasing the signal and the noise by the same proportion amounts to multiplying both sides of Equation 2 by the same factor, which would obviously not change the LOD. However, increasing the resonance amplitude (first term in Equation 2) by reducing the losses does not lead to increasing the middle term ($A(\lambda')$) by the same factor. It is this feature that leads to the different powers between noise and signal in Equation 4.

The importance of the resonance amplitude can be further appreciated by comparing the dependency of the LOD on the SNR in systems with and without losses. For this comparison, we use, on one hand, our model (Equation 4) assuming fixed noise and varying the resonance amplitude (red curve in Figure S2); on the other hand, we use Fan and White's model [12] to describe the LOD dependence on the SNR for a lossless system (blue curve in Figure S2). We assume $3\sigma = 1.42\text{x}10^{-2}$ and the $Q_R = 540$ which are the parameters used for the validation of the model in Section 2.2 of the main text. A comparison between the plots shows that the LOD deteriorates much faster with the SNR when losses are included, which is a consequence of the different powers between σ and $A(\lambda_0)$ in Equation 4. Such a fast deterioration emphasizes the importance of the resonance amplitude in defining the LOD.

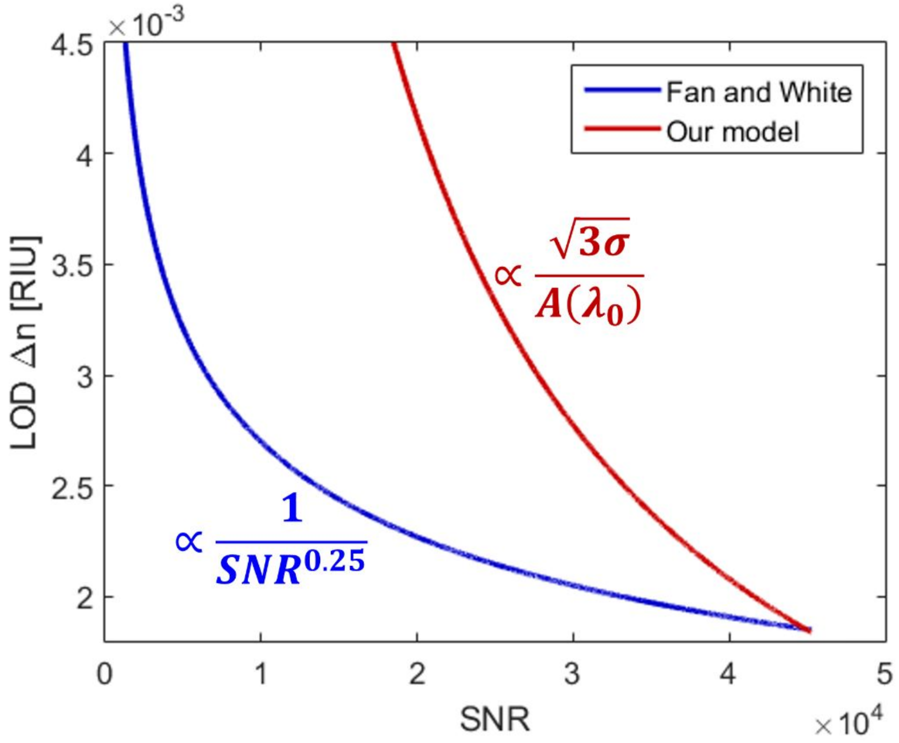

Figure S2: Comparison between our model (red curve), which takes losses into account, and Fan and White's model (blue curve), which does not take losses into account. When losses are included, the SNR is no longer a key parameter describing the LOD, and it becomes more important to increase the signal than to reduce the losses. The red curve is generated assuming $3\sigma = 1.42x10^{-2}$, which is the measured value used for the validation of the model in section 2.2.

Supplementary Information 5

Sensitivity of the GMR sensor

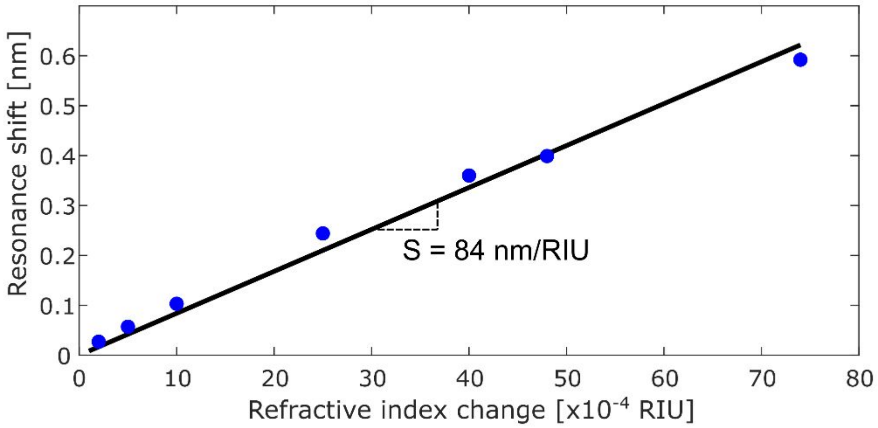

Figure S3. Resonance wavelength shift for different values of refractive index change measured with a GMR structure with Q_R = 540, $A(\lambda_0)$ = 0.59 and λ_0 =743 nm, obtaining a sensitivity S = 84nm/RIU

Supplementary Information 6

Resonance amplitude changes of a GMR sensor with higher Q_R but lower $Q_R A(\lambda_0)$

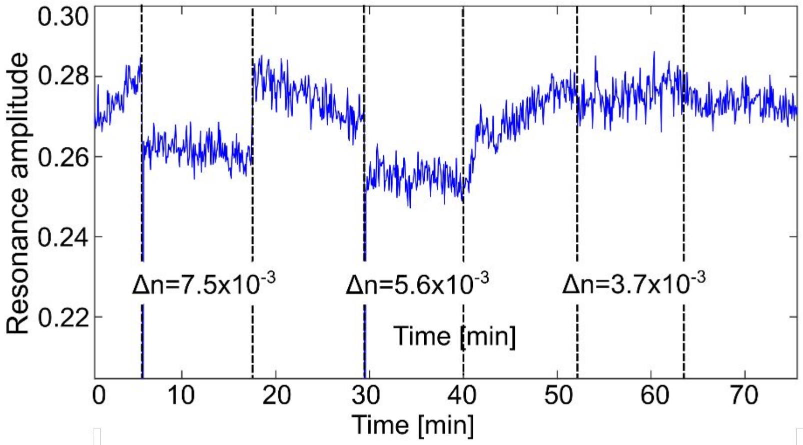

Figure S4. Resonance amplitude change over time for different refractive indices. This specific GMR sensor used here has the parameters Q_R = 890, $A(\lambda_0)$ = 0.27 and λ_0 =743 nm.

Supplementary Information 7

Effect of data fitting

We use the method to compare the LOD obtained with and without fitting. The resonance wavelength was extracted by fitting the experimental curve in Figure 2a with a Lorentzian curve, using the method of nonlinear least squares [28]. Figure S5 shows the resonance tracking of the same sensor. As is apparent from the curve, this simple procedure reduces the LOD to a value of $LOD \leq 2\text{x}10^{-4}$ RIU. We note that even lower limits of detection may be obtained with more efficient fitting methods or by oversampling the data [27, 29-30], so the fitting method used here only serves as an example. Using this method, we extrapolate the equivalent value of 3σ from the model that would be required to provide this low LOD without any fitting procedure. Using Eq. 4, we find that the equivalent noise is $3\sigma = 1.4\text{x}10^{-5}$, which is three orders of magnitude lower than the raw noise. We conclude that, by employing a simple fitting procedure, one obtains the same LOD that would be obtained in a system with a standard deviation of the noise that is a thousand times lower.

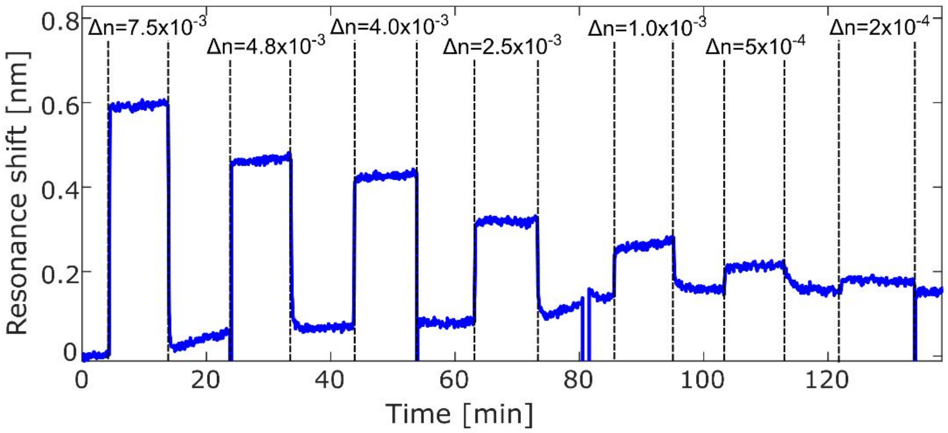

Figure S5. Resonance shift over time with fitted data for different values of refractive index of the solution.

Supplementary Information 8

Dynamic range of Fano resonances

The expression for a Fano resonance can be obtained from Temporal Coupled Mode Theory. Again, considering a single resonance coupled to two channels, the Fano resonance is given by [18]:

$$A(\lambda) = \left| r - \frac{Q_R^{-1}}{2i\left(\frac{\lambda - \lambda_0}{\lambda_0}\right) + Q_R^{-1} + Q_{NR}^{-1}} \left(r \pm t\right) \right|^2 \tag{S21}$$

Where r and t are the reflection and transmission coefficients, and the symbols of plus or minus depend on the symmetry of the resonance [17]. Assuming a symmetric Fabry-Perot background resonance, r and t are given by 18]:

$$r = \frac{(1 - e^{-i\delta})\sqrt{R}}{1 - Re^{-i\delta}}, \qquad t = \frac{(1-R)e^{-i\frac{\delta}{2}}}{1 - Re^{-i\delta}} \tag{S22}$$

Where R is the single interface reflection coefficient. Notice that $\delta = 2\pi$ results in $r = 0$, and $t = -1$, which is the condition for a pure Lorentzian as discussed in Supplementary Information 1 above.

Plots of $A(\lambda)$ for different δ are shown in Figure 4. In all cases, the dynamic range is determined by the separation between peak and dip. Notice that the resonance for $\delta = \pi$ is mostly a dip resonance. It is this feature that contributes to its larger dynamic range.

To better understand the origin of the higher dynamic range, the relationship between the resonance and background amplitudes and phases are shown in Figure 4. The resonance is represented by the term $A_c(\lambda)$, where

$$A_c(\lambda) = -\frac{Q_R^{-1}}{2i\left(\frac{\lambda - \lambda_0}{\lambda_0}\right) + Q_R^{-1} + Q_{NR}^{-1}}(r \pm t) \tag{S23}$$

while the background is given by r. This latter term represents a direct pathway, which can be understood as the portion of the light that is reflected directly, without coupling to the resonance. As shown in equation (S23), the amplitude results from interference between r and $A_c(\lambda)$.

As shown in figure 4, as the resonance evolves from a pure Lorentzian ($\delta = 2\pi$) towards a Fano ($\delta = \pi$), $A_c(\lambda)$ tends to align with the inverse of r. This alignment promotes a full destructive interference between the two pathways, thus softening the reduction of the $A_c(\lambda)$ amplitude due to the losses. Consequently, losses affect the resonance dip less for the case of a Fano resonance ($\delta = \pi$) than for the case of a Lorentzian resonance ($\delta = 2\pi$).
